# Bivs, Space and ‘In’

**DOI:** 10.1007/s10670-019-00198-z

**Published:** 2020-02-11

**Authors:** Clare Mac Cumhaill

**Affiliations:** Durham University, Durham, UK

## Abstract

I present a novel anti-sceptical BIV argument by focusing on conditions on the production and use of the locative preposition ‘in’. I distinguish two uses of ‘in’—material and descriptive phenomenological—and I explain in what respect movement is central to the concept that our use of ‘in’ expresses. I go on to argue that a functionalist semantics of the intelligible use of ‘in’ demands a materialist philosophy of action in the spirit of G.E.M. Anscombe, but also why the structure of space is not irrelevant either; appeal to the structure of space unsettles the causal-empirical assumptions that ground the picture of subjectivity and agency that the biv narrative assumes. Finally, I explain why a functionalist semantics demands a Naïve Realist metaphysics of perception, consistent with some of Putnam’s last writings on philosophy of perception.

## Preamble

There are different ways of formulating Putnam’s notorious antisceptical ‘biv’ argument, just as there is diverse opinion as to whether it succeeds. I will assume that it does in the following form: My word ‘brain’ refers to brains, the biv’s word ‘brain’ refers to brains*, and the same goes for vats and vats* and for thoughts as well as words. Thus, were I a biv, the thought that I could be such would be *unrepresentible*—my thoughts and words refer to brains* and vats* not brains and vats. This is a formulation of the argument in Kantian guise.^1^ In this paper, I make a different antisceptical move, one that, so far as I can tell, has not yet been tried out.

The above formulation is fairly generous to the biv since it grants that the biv’s words and thoughts refer, that their referents can be ostensively picked out, and their thought contents are individuated externally; just as I can point to trees, so the biv can point to trees*; just as my thought that oaks spring from acorns gets its content through my being causally related to oaks, perhaps at various times, the biv analogue gets its content through the biv being causally related to whatever plays the role of ‘oak’ or ‘acorn’ in the envatted world and its respective seasons*.^2^

The argument I propose is less generous. The standard formulation grants that a biv can conceive of itself as a brain* in a vat*. One lesson of the paper is that we should append a star too on the locative preposition ‘in’. But the locative is challenging in a way that oaks and acorns might not seem to be. First, there is no one recurrent visual feature that ‘in’ picks out. As I explain, the intelligible use of ‘in’ more often relies on a grasp of the nature of the relata it relates, but there is no one kind of relation that ‘in’ names and that is repetitively instantiated wherever the use of ‘in’ intelligibly applies. Second, movement is central to a grasp of the concept that the use of ‘in’ expresses. However since the possibility of movement is sensitive to the structure of space—and here I mean the unimpeded translation from one place to another of things like purses, bikes and birds, as well as our own movement—the structure of space in our vicinity is explanatorily relevant to the intelligible use of ‘in’. Absent that structure and it is not clear what the content of ‘in’* could in fact be.

I think there are a number of interesting consequences that follow from this way of taking up the biv argument, but here I plumb only one.

It has been assumed that the later Putnam was wrong to think that he needed a particular philosophy of perception to stem the sceptical worries that come from applying arguments derived from model theory to experience, worries that the biv argument was supposed to allay.^3^ For instance, Tim Button, whose *The Limits of Realism* sets out these arguments beautifully, has it that so long as experience veridically represents how things are, reference is secured, and so long as reference is secured, the nature of our perceptual access to the world is irrelevant to the success of the biv argument. The appeal to perception is “a red herring” (2013, p. 92).

I think that focus on the locative ‘in’ shows that this is not so, and to develop this idea I draw on a remark made by Putnam more than thirty years later, in a paper delivered in 2012.^4^ Putnam recruits G.E.M. Anscombe, albeit in parenthesis:

There are apperceptions that have *no* accompanying qualia at all. Suppose I raise my right hand. My awareness that *I raised it* (it didn’t simply “go up”), is a genuine awareness, a genuine act of apperception, but there is no *quale* of voluntariness. (I think I remember that Elizabeth Anscombe somewhere describes this kind of awareness as “knowledge without observation”, but this seems to me to be a misdescription. I would say that I *did* observe that I raised my hand, but this is observation without any particular qualia).

A you-tube video of the original talk shows Putnam raising his arm. But rather than consider what Putnam says, I focus more on what he does—*move*—though I do pick up, and make central, his only passing appeal to Anscombe.

Anscombe develops what Ford (2018) calls a *materialist* philosophy of action, something that, as I show and explain, a *functionalist*-*geometric* treatment of the semantics and use of ‘in’ requires. At the same time, acting on the material world typically involves passage or movement through space—*simple movement*, call it. Such simple movement is sensitive to the structure of space in ways I (very generally) explain. But since this is so, there is a connection—if granted a non-obvious one—between the shape of space, and the human concept which a capacity to use the locative preposition ‘in’ expresses.

Why should any of this matter for a metaphysics of experience? As I explain, the conception of experience that the biv argument relies upon not only forecloses theoretical recognition of the *materiality* of action—the fact that we act on and are directly responsive to purses and bikes and not to causal input delivered by impact from these things—it concomitantly removes from the province of explanation the space in which our activity unfolds, the structure of which our simple movement is sensitive to. But it is against this arena that a Naïve Realist metaphysics of experience can, I think, be brought into theoretical purview. And this is because, as we shall see, the way in which space explains is not in respect of its being efficient, but in respect of its structure.

This is a hard argument to make, mostly as it tries to hang in the balance two ‘aspects’ of philosophy that might otherwise be seen as at remove from each other: the form of human action (compare the breakfasting Moore) and the structure of the space in which, on a grander scale, it unfolds. The argumentative strategy of the paper is nonetheless to circle back from one aspect to another. It unfolds as follows: I note two ways in which the word ‘in’ is relevant to a formulation of the sceptical scenario and I show that we ought to prefer a functionalist-geometric treatment of its semantics over an abstractionist or ‘ideal’ geometric analysis. I introduce the concept of *locational control* and I set out how we might understand the notion of ‘function’ above, making contact with Putnam’s move to Naïve Realism in the mid-Nineties. Finally, I sketch the argument that links our grasp of ‘in’ to a Naïve Realist theory of perception, as well as an objection, where here, again, Anscombe will come to the rescue.

## Two Uses of ‘In’

Putnam’s biv is in a vat, tulips are sometimes in vases, pennies in purses. Let us call the *material* use of ‘in’ that use which describes object-object relations of this sort and distinguish this use from a *descriptive phenomenological* use which captures the relation that everyday objects appear to bear to the space of our experience—they appear to be ‘in’ or ‘within’ it, just as bits of furniture occupy a room. Here’s Michael Martin exemplifying this latter use:

[T]here is in normal visual experience some sense of not only being aware of objects *in* space, but being aware of a region of space they occupy….one can have some sense of the space that objects are located *in*, even when that space is not itself completely illuminated, or is partly obscured. Shining a torch across a room illuminates in turn different parts of the room and objects *within* it, but this does not necessarily alter the field of vision; rather there is a sense of different parts of the scene being lit up. In the same way, the rearrangement of objects so as to reveal what was once obscured seems to be a rearrangement of objects within a space experienced rather than an alteration in that space (Martin 1992, p. 214).

I pick up on the notion of ‘rearrangement’ of objects in space a little later.

Now, there is no antecedent reason to suspect that these two distinct uses mark out different senses—indeed, on my view, they are part of the structure of the same concept. Nonetheless, it is useful to distinguish them for two reasons. Firstly, it is plain that a putative biv subject that can conceive of the sceptical scenario in the form envisaged by Putnam should have a grasp of both uses. The biv must conceive of itself as a brain in a vat (the *material* use). But the conceit also has it that the biv has experience *just like us*. Accordingly, *its* experience should be captured by the kind of descriptive phenomenology that Martin offers—that is, the biv subject ought to have phenomenology that can be captured by the descriptive phenomenological use. Such a subject should be aware of objects ‘in’ space, located at places.

The second reason is the following. On an empiricist conception of experience whereby it is supposed that what is ‘given’ to one is little more than, in the visual case, a colour mosaic (say), the possibility of wringing a conception of the ‘external’ world from such bare sensory experience—and, so, for some philosophers the possibility of a subject even having the phenomenology Martin describes—requires explanation. In his justly canonical (1985) ‘Things Without the Mind’, Gareth Evans urges that what is needed is a conception of substance. His master argument is to show that only a world that contains space-occupying substance (for him substance of the sort that can ground a naïve physics) can allow for the possibility of a subject’s having a conception of persistent substance in the first place. I don’t explore Evans’ transcendental argument here, but there is a sense in which one wing of the argument I offer has a parallel structure, and this is so despite revoking the ‘empiricist problematic’ which asks for an explanation as to how it is possible that, on the basis of fragments of sensory input, we can have the conception of an external world at all. I argue that simple movement is sensitive to the structure of space and that, as such, the structure of space is relevant to a grasp of the concept which the locative ‘in’ expresses (this, then, is the strand of thought that chimes with Evans’ transcendental strategy). But since the structure of space is relevant in this way, this brings into our explanatory purview a form of explanation that is non-causal; after all, it is not in virtue of anything that space *does* that brings it about, in an efficient sense, that movement through it is sensitive to its structure. And this in turn explains why appeal to the structure of space is challenging for friends of envattedness, as well as why the ‘empiricist problematic’ is, in the end, out of place on view I set out to develop. But early assimilation of the material and descriptive phenomenological uses makes it hard to bring reasons for the rejection of assumption of the empiricist problematic into view.^5^

## The Material Use and Locational Control

I have so far distinguished two uses of ‘in’. In this section, I want to say something about object-object ‘in’ relations in particular. This is since even brief consideration of these shows that any kind of hope for an *abstractionist* account of the acquisition of the concept that ‘in’ expresses should be left aside at the outset.

Here are four observations concerning the material use. Where such a use is licenced, the contained object is typically *smaller* than the container—the penny is smaller than the purse, the brain is smaller than the vat. Second, where the object is immersed in or enclosed by the containing object, it makes sense to say that neither are *path*-*connected*. If two points are path-connected, they are in the same space—there is path through space that connects them. If one object is enclosed by another object however, the contained object is in the space *of the container*. Both do not share the same space (think of Russian dolls).

Path-connectedness is a topological feature that defines a space. But while topology is an abstract art, humdrum reality is different. Take a closed biscuit-tin and a child who is trying to prise it open. There is a path through space that connects the child’s fingers to her treasure, but human fingers cannot move through tin. If the biscuit tin were open, things would be different, but we would still maintain that the biscuit is ‘in’ the tin; the locatedness relation that ‘in’ expresses is consistent with *partial* enclosure. Third, the contained object is often, though not always, *mobile* relative to the container—we say that the bird is in the tree, that teeth are in mouths. Teeth can fall out but typically we hope them to be immobile. Finally—and importantly—the container typically exercises *locational control* on the contained.^6^ I spell this idea out since it will be useful later.

**Fig. 1:**
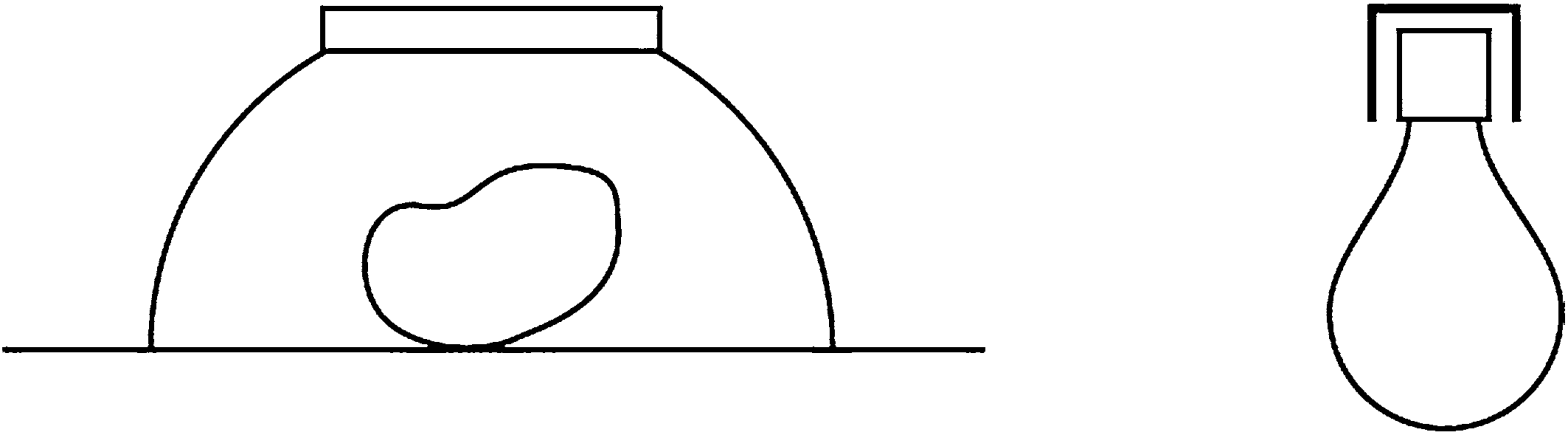
From Herskovitz (1985, p. 354)

When a container is moved, its contents typically move with it. The container determines *where* the contained is located. Call this *weak* locational control. As an illustration, consider what looks like a potato in the upturned bowl on the left, below. If the bowl is moved far enough in either direction, the potato will move too. Suitably understood, then, the potato is ‘in’ the bowl. But matters are not always so straightforward. While we can happily say that the bulb on the right is in the socket, it might, on occasion, be *less* permissible to say that the potato is in the bowl. This is because as things now stand with the bowl, the bowl is not currently exercising *strong* locational control on the contained, call it. In cases of strong locational control, as I will understand it, the shape or structure of the container constrains the possibilities for movement of the contained. Because the potato is resting on the table in Fig. 1 however, the shape of the bowl is not exercising strong locational control on the potato.

Simply listing these four features shows that our use of ‘in’ has a degree of complexity—and this is leaving aside all metaphorical uses. But not all dimensions figure in any given token use, though two or more often do. We might then reasonably ask whether entailments flow between certain conditions, or less rigidly, features. For instance, the fourth characteristic feature might seem to entail the first, though this is often not the case. Flowers can be in a vase, but usually only the stems are ‘immersed’ in the interior volume of the vase and the water it contains. Nevertheless, the vase exerts locational control on the flowers. Likewise, though locational control might seem to entail the second characteristic feature—relative mobility—sometimes, the control, we might say, is absolute: the contained is held fixed, as the bulb in the socket is. In certain cases however, locational control might not seem to apply in such a strict sense at all, as, for instance, on a certain reading of ‘the bird is in the tree’. Birds fly. Even so, it remains the case that there is a peculiarly *avian* way for birds to be in trees, one that is distinct from ways in which squirrels, say, characteristically occupy or are ‘in’ trees.^7^ That this is so suggests that there may be a *normative* respect in which we can conceive of locational control. It may be ordered or disordered; the structure of the places in which creatures find themselves, including their larger environs, can shape ongoing patterns of activity in ways that are, or are not, characteristic of their species or kind. When a bird is caged, the locational control the cage exerts is disordered. Of course, the same is true of the human brain envatted.

Without exploring this or detailing these interdependencies any further, we can I think grant that the conditions that invite us to say that something is ‘in’ something else can vary enormously, though there is often a pattern in our use which we can trace in particular cases and which may deviate more or less from other everyday uses. But if this is right, this suggests that we should resist anything like a straightforward definitional account of the meaning of in—one that recommends necessary and sufficient conditions for its appropriate application say. That is to say, we should not maintain:

1. In (*X*, *Y*), iff Located (*X*, Interior(*Y*)) (cf. birds in trees, cracks in jars).

Nor should we hazard anything like the following:

2. IN: *X* in *Y*: *X* is located internal to *Y*, with the constraint that *X* is smaller than *X*—where *X* is the located object and *Y* is the reference object (Cooper 1968) (cf. flowers in vases).

Or

3. IN: *X* in *Y*: *X* is “enclosed” or “contained” either in a 2D or 3D place *Y* (Leech 1969) (cf. open biscuit tins, birds in trees).

But this is not all. What brief reflection on this range of cases shows is that, *contra* an ambitious concept empiricism, there is, in the case of ‘in’, no one recurrent visual feature present in all cases which could tempt the suggestion that we *abstract* from *those* visual features to acquire the concept which the use of ‘in’ expresses. Likewise, nor is it the case that the presence of any such recurrent feature warrants the application of the locative on some occasion.^8^ Cracks in jars are very different to both birds in trees and bulbs in sockets. Rather that such different objects, including even absences, can figure as contents and containers suggests instead that our production and intelligible use of ‘in’ is responsive to some other constraint or constraints. This is what I explore now by setting out one influential approach to tidying up this variation.

Herskovitz (1985) suggests that there in fact is an ‘ideal’ meaning of the locative expression ‘in’ around which others gravitate. This ideal meaning is characterized as ‘the inclusion of a geometric construct in a 1- 2- or 3-dimensional geometric construct’ and covers an impressive range of use types: The crack, for instance, is a one-dimensional geometric construct in a two-dimensional surface and the bird is a spatial entity in the three-dimensional environment of the tree. Importantly, this ideal meaning approach prioritizes abstract *geometric understanding* over any kind of understanding of the nature of objects involved, but we might expect this to be problematic for two reasons. First, the emphasis on ideal meaning, where this is construed geometrically, often fails to get at why using locatives has a point. When we say that something is ‘in’ something else, we typically don’t mean to give a mere ‘snapshot’ reckoning of where something is at a time. Rather, we aim to supply information about how, that thing being so contained, we expect things to unfold over time. Yet how things unfold over time depends on the nature of the container and the contained. Biscuit-tins are a designated place for biscuits but being told that some biscuits are ‘in’ some tin is typically in response to a *question*, the asking of which, presumably, has consequences for their enduring containment. Trees are the proper resting places for certain kinds of nesting birds. Being told that a bird is in a tree however, might seem to have no consequences for its containment in the tree over time, *assuming* that is that the relevant containment and locational control is ordered—so, it is not the case that it’s leg is *caught* in a branch. Compare the princess with the glass heart. Suitors had to treat her carefully so that her heart would not break.

**Fig. 2:**
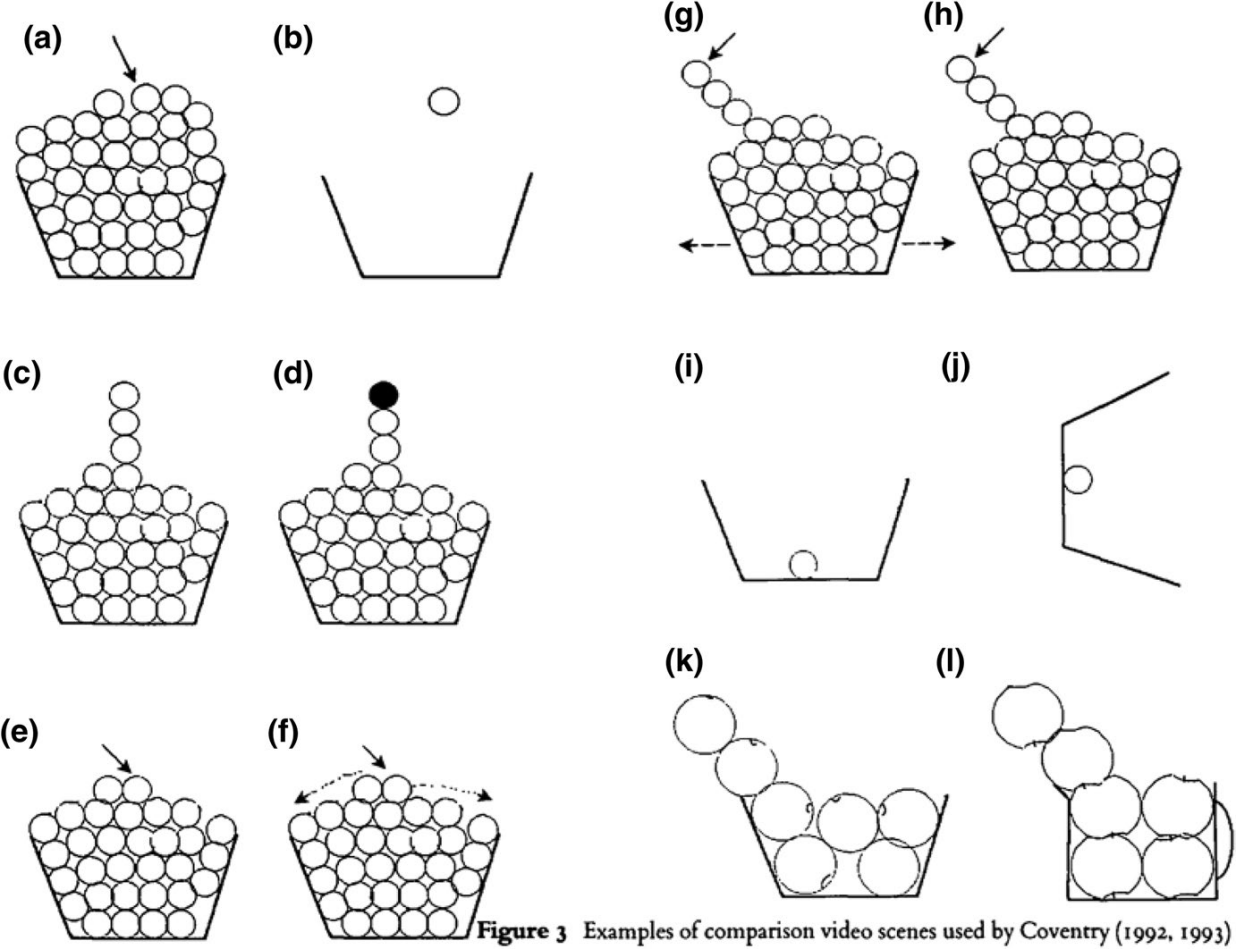
From Coventry, Carmichael and Garrod (1994, p. 294)

Second, unlike the topological relation of inclusion noted earlier, a functional understanding which takes due account of the natures of the *relata* involved is not transitive. If an umbrella is someone’s hand, and their hand is in a glove, the umbrella is not also in the glove. Where an abstract topological treatment licenses certain geometric or spatial inferences, the world does not. As we have seen, further, sometimes evidence of weak locational control is sufficient to license the use of ‘in’ and in such cases the geometry of the container is hardly relevant at all. For instance, consider the pattern of Fig. 2.

The arrows pointing outwards in (g) symbolize movement. If the object in (g) moves *with* the bowl it is ‘in’; the item is judged to be ‘in’ the bowl. Conversely, if some item moves independently of the movement of its supposed container, ‘in’ is less likely to be applied, as in (f); in (g) the object pointed out by the arrow is more likely to be judged to be ‘in’ the bowl than the item in (h).

Function and orientation play a role too. The item at the top of the pile in (l) is less likely to be judged ‘in’ the container (a jug) than the item at the same position in (k) (a bowl), while the object in (i) is more likely to be judged ‘in’ its container than (j). (b), finally, does not appear to be ‘in’ the bowl at all—locational control would fail here over time, miracles aside.

Such considerations suggest reason then to favour a *functional*-*geometric* approach to the production and intelligible application of ‘in’ over an ideal geometric approach.^9^ A functional-geometric approach is one that takes due notice of the natures of the relata (a jug or a bowl?) as well as the naïve physical intuitions that seem to inform locational control—and, as we have seen, where one or more of *relata* is a living thing, there are further considerations to bring to bear, considerations that are important in ways that I cannot explore in this paper. Instead, in the next section, I develop a rather unexpanded notion of the idea of a ‘function’, though one that will suffice for my limited purposes here. In particular, I begin spelling out how we can connect observations concerning the semantics and use of ‘in’ to Putnam’s later suppositions as developed in his Dewey lectures that a particular *philosophy of perception* is needed to stem the sceptical worries that his earlier biv argument was supposed to allay. To pre-empt that discussion, here’s a glimpse at the connection:

In order to accommodate, at the perceptual level, what the functional-geometric approach seems to require—namely, that a grasp of the natures or kinds of candidate containers and their contents is relevant to a consideration of whether the production of ‘in’ is appropriate on occasion—it might be thought it need only be insisted that experience is *rich* enough to represent the functional and geometric dimensions that are relevant to warranting the application.^10^ For instance, even if it is insisted that the admissible contents of experience are narrow,^11^ or if it is maintained that the phenomenal character of experience is constituted by observational properties like colour and shape, so long as experience (however this is to be theoretically understood) can warrant the application of the relevant kind concepts and sortal terms to the world (‘jugs’, ‘cages’, ‘pockets’), there is no reason to suppose that offering a functionalist-geometric semantic treatment of the production and use of ‘in’ should necessitate any particular philosophy of perception at all. But, if so, even with respect to ‘in’, perception is—as Button supposes—a red herring. As will become clear, I disagree but the route to getting to that conclusion is not direct. It involves first an exegetical detour and then an extrapolated appeal to philosophy of action. Hopefully the reader will bear the course.

## Apples and Fields: Two Notions of ‘Use’

So, is it the case that endorsing a functional-geometric conception of the use and semantics of ‘in’ necessitates a particular philosophy of perception? To approach an answer (something that will carry us into the next section), we need to distinguish two notions of *use* that the self-reflective Putnam of the Dewey lectures sees at work in his own writings—and here, for expediency, I assume a little bit of familiarity of the part of the reader.

The Putnam that introduces the biv argument is an *internal* realist. For the internal realist, unlike her external counterpart, the way our words are responsible to reality is not fixed; words like ‘brain’ make sense *within a theory*, hence the *internality* of the realism involved. Even so, words manage to ‘hook’ onto reality. The relevant yoke is causal-referential. It involves naming, which itself involves ostension and so the ‘intimate connection’ of *perceiving*:

We are able to perceive, handle, deal with apples and fields. Our talk of apples and fields is intimately connected with our nonverbal transactions with apples and fields (Putnam 1981, p. 11).

For the Putnam of the Dewey lectures however a new gloss on the semi-technical idiom of ‘transaction’ above is needed, as well as on the ‘intimate connection’ of perceiving. In his earlier writings, the notion of ‘transacting’ was conceptually linked to a Wittgensteinean notion of use, albeit one that was routed through machine-state functionalism:

The notion of use that I employed….was a “cognitive scientific” notion; that is, use was to be described largely in terms of computer programs in the brain…. There was the computer program in the brain, and there was the description of the external causes of the language user’s words (1994, pp. 457–458).

But it is this conception of *use*—“a portmanteau affair”—that the Putnam of the Dewey lectures now seeks to repudiate. This is since despite his Wittgensteinean appreciation of the importance of use to a theory of understanding and meaning, his earlier metaphysics of mental functioning remained, by his own later reckoning, “a “Cartesian-cum-materialist” affair, a picture of mental functioning on which it seems “magical” that we can have access to anything outside our “inputs”. He explains further:

To the extent that I was aware of something that could be called “direct realism”, the “direct realism” of which I was aware was only the superficial linguistic reform that does nothing except to make a verbal modification in the way the traditional picture is presented. If one holds that traditional picture fixed, as I did, then the verbal modification (the modification that consists in allowing that we can say we “observe” external things, but of course that must be understood as meaning that those things cause us to have certain “qualia”, and that they do so “in the appropriate way”) seems, at bottom, just a way of hiding a problem, the problem of how even our perceptions can be determinately of particular external things… (ibid., p. 464)

and the problem, if hidden, remains, since, despite linguistic reform (the reform the semantic externalist recommends), once a certain conception of experience persists, one whereby experience is understood to be veridical if caused “in the appropriate way”, there is nothing to stave off the possibility of experience assuming its age-old status as a ‘veil’ through which the world is perceived, a world which is thereby only an approximation of what’s ‘out there’—different worlds can bring out the same experience.

Presciently then, the Dewey-inspired Putnam advances a wholly familiar and ‘natural’ notion of use, one on which our transactions with the world do not have the sterility of ‘input’ after all and ‘reach all the way’ to the apples themselves, to the fields we are ‘in’. With these two notions of use distinguished, we are now in a position to ask whether the notion of *function* behind the geometric-functionalist treatment of the semantics of ‘in’ lines up with either of these notions of *use.*

Since functionalist-geometric approach revokes any kind of abstractionist account of the acquisition of the concept of ‘in’, it seems that we should straightforwardly resist a conception of use whereby the visual system ‘uses’ certain stimuli patterns as ‘input’ to license the application of ‘in’ on occasion. As we have noted, the functional-geometric approach emphasises how *things* are used in a sense that takes note of their purpose and nature, something that, prima facie, seems more in line with Putnam’s later notion. Yet even if this is so—namely that functional-geometric approach is, in this sense, more ‘natural’ let’s say—why think that a particular philosophy of perception is needed to so as to make intelligible, indeed possible, what we are assuming is the most plausible account of the semantics and use of ‘in’? It is very hard to see why it should be! Shifting to the kind of philosophy of action implicit in Putnam’s (1981) thought experiment helps us get a little further.

## Putnam’s Gesture

In the youtube video of the 2012 talk mentioned at the outset, we see Putnam raising his arm. According to the Putnam of *Reason, Truth and History*, such gestures occur too in the vat:

There seem to be people, objects, the sky, etc; but really all the person (you) is experiencing is the result of electronic impulses travelling from the computer to the nerve endings. The computer is so clever that if the person *tries* to raise his hand, *the feedback from the computer will cause him* to ‘see’ and ‘feel’ the hand being raised (my emphasis, Putnam 1981, p. 6)

Let us consider the import of the italicised terms. With the use of the word ‘tries’ Putnam seems to implicitly endorse what Anton Ford has recently dubbed a *volitionist* philosophy of action. Such an account gives rise to, as Ford puts, a ‘practical correlate of the argument from illusion’. On a volitionist treatment, what is primary is a volition or a trying or willing, where what happens after that is ‘up to the world’. On this view, there is a gap between willings and their effects. In the good case, the world co-operates and we succeed in doing what we were trying to do. In the bad case, it does not. Either way, for such a theorist, *where the action is* in the trying, where this—a *trying*—has been reified into some kind of mental entity, one that, like a sense-datum in the perceptual case, is the highest factor that is common to both a successful trying and to one that is not. Yet notice here what successful willing in the vat amounts to: a conjunction of phenomenal experiences that we may be apt to describe as ‘seeings’ and ‘feelings’ *of a hand being raised*. I return to this specification later.

Now, since the Dewey-inspired Putnam repudiates a model of perception whereby the closest the perceiver gets to the world is by way of “input” delivered to his sensory surfaces, we should expect a rejection of the input-output model in the case of action too, and Putnam’s emphasis on *trans*action, which might be thought to involve an exchange or transfer, chimes with this ambition. But since we now know that what is exchanged or transferred is *not* now to be understood in terms of input and output, how should we understand this notion?

It is a reasonable hermeneutical guess to suppose that what Putman must have in mind is a philosophy of action that models Naïve Realism in the domain of perception. That is, it might be thought that for the Naïve Action theorist, as for the Naïve Realist about perception, action is essentially *world*-*involving*. As we will see shortly, however, this bare analogue needs careful elucidation. To see why, we need to distinguish the Naïve Action theorist not only from the volitionist but—again borrowing Ford’s taxonomy—from the *corporealist*.

For a corporealist, what is theoretically privileged against willing is bodily movement *tout court*. But, on this view, the kinds of actions that we might consider world-involving—pretty much any human action you care to mention, all “the acts by which human beings sustain human existence” as Ford puts it (walking, eating, copulating)—are “second-class expressions of human agency” (p. 710). Rather it is primarily *by moving its body* that an agent is understood to indirectly cause a change in extra-corporeal objects, including presumably in other people. Like the volitionist then, the corporealist also falls into a kind of practical dualism, though the boundary of where the action is extended now outside putative willings to the *body*.

Now, this, plainly, is not what Putnam has in mind with his talk of reaching ‘all the way’ to apples and fields. As a methodological heuristic, we might then look for specific analogues that would draw the domains of perception and action closer. For instance, we might look for the action analogue of the Naïve Realist claim that the contours of conscious character are shaped by the objects, and their intrinsic properties—their shape for instance. With this in mind, consider Ford:

In the ordinary course of practical life, bodily movement does not have a contour of its own: it takes the shape, liquid-like, of what we pour it into. By way of analogy, consider that if one poured water from pitcher over two differently-shaped objects arrayed side by side—e.g., a rod and sphere—the water would move differently as it fell upon the objects, and over and around them; and the difference would be explained by the shape and orientation of the objects. Similarly, if one were to *grasp* each of these objects, there would be differences in the way one’s hand closed down around them; and the differences would be explained, again, by reference to the objects. For, one grips a rod differently than a sphere; and how one grips a rod differs according to how the rod is oriented with respect to one’s body. The most important disanalogy between the movement of the water and the movement of one’s hand is that, between the lip of the pitcher and its contact with an object, the water falls in the same way no matter what it falls upon. By contrast, the movements one makes in reaching out for an object differ according to the grasp that one must ultimately take of it. (p. 708)

This passage undermines the idea that, although differences between grasping the rod and grasping the sphere can be explained by *reference* to differences in the objects themselves, the movement of the body that grasps them is such that *it* can be cleaved apart from the objects—the rod and sphere—that, on such an assumption, *give rise to them*. Instead, like the Naïve Realist claim that conscious character is shaped by the objects and their intrinsic properties that the perceiver is aware of, it might be thought that the contours of bodily movement are *constitutively* shaped by the objects, and their intrinsic properties, that the agent is acting on or transacting with. Yet this parallel remains inadequate—and this connects back to Putnam’s reflections on the second notion of use above. It also requires us to take seriously the Naïve Realist assumption that experience is not world-dependent but world-*involving*; a claim that is distinct though related to the thought that the fundamental nature of phenomenal character is such that it is constitutively shaped by the worldly objects and their perceptible properties.

The quoted passage (though not Ford’s paper as a whole) abstracts from the fact that such ongoing transactions are typically intelligible in the context of *wider action wholes* of which the current transaction is a part. These wider action wholes are thereby *involved*. Think of the intricate finger-work involved in tying laces. While the contours of the bodily movement of a particular lace-tying event are constituted by and inherited from the particular shoe-laces being tied by a particular historical individual at a time, tying one’s shoelaces is typically part of a wider action whole, a whole in which it is thus involved. For instance, I might be tying my shoelaces because I’m going to the shops to buy butter to bake a cake for a visitor I am expecting.

So, think now of typical transactions with apples and fields: picking, peeling, stewing, hoeing, and harvesting. The contours of the bodily movements involved in all these actions are constituted by the properties of the apples picked and peeled and the fields hoed and harvested at any particular time. Picking is an elementary action, peeling slightly less so, but hoeing and harvesting, the transactions *they* involve, are quite obviously only intelligible in the context or larger action wholes of greater or lesser temporal extent—the keeping at bay of otherwise invidious greenery so that seeds can grow, grain milled and, after a time, cakes baked.

Ford calls the picture of human action that appeals to the wider action wholes in which worldly constituents participate *materialism*. On this view, action does not stop at willing or at the boundary of the body but goes all the way to the material, the apples and fields, the subject is transacting with, and whose transactions are intelligible in the context of wider, spatiotemporally extended action wholes in which that material participates, in which they are thereby also involved and which fall under standard descriptions: ‘hoeing’, ‘harvesting’, ‘baking cakes’.

This synopsis is brief in the extreme. Still I think it is enough to grant the following: A functional-geometric account of the semantics and use of ‘in’ is more at home in the conceptual company of such a materialist philosophy of action than one that is volitionist or corporealist—after all, the functionalist-geometric account emphasises knowledge of what the container and contained are and are *for*; how they are to be used and for what ends. That is why, after Anscombe, the materialist draws attention to the manifold different ways in which we, agents, do things, ways that are captured by causative verbs, e.g. “*scrape, push, wet, carry, eat, knock over, keep off, squash, make* (e.g. noises, paper boats)*, hurt*” (1981, p. 137). But naturally we can also add locative verbs to this list, e.g. stuff, dump, heap, load, pour, fill. wipe, rid, drain, clear, remove, empty. Certain of these pick out a change of state of the container (it is filled, stuffed heaped loaded with/by); some pick out a change of state of the contents of the container (they are heaped, loaded, poured into/onto etc.); many containers have their particular *modes* of being filled: we stuff suitcases, load carts, heap bowls.^12^

What is the significance of all this? Broadly, the idea is that stuffing, loading, heaping are actions that agents perform or simply do in the normal course of going about in the world—a materialist insight. But learning which things can be permissibly stuffed is to learn something about the kind of thing stuffed and its function—stuffing a suitcase is fine so long as you can carry it. These considerations suggest it is right to pair the functional-geometric account with a materialist philosophy of action. The suggestion too is that the notion of ‘use’ in play is at home with the conception of use espoused by the later Putnam. But if this is right, ‘reaching all the way to the apples and fields’ turns out to be a *condition* on an adequate grasp of the appropriate use of ‘in’. What about a philosophy of perception? Earlier, I promised that a detour into philosophy of action could help spell out lessons in this domain too. In the next part of the paper, I explain how so—and here the concept of locational control will be harnessed, albeit beyond the homely scale of Moore’s breakfast bowl. In conclusion, I tie together both aspects of my argument—appeal to the form of human action and to the structure of space.

## Movement in Space

In his ‘Introduction to Anscombe in Context’, Fredrick Stoutland notes the respect in which Anscombe’s outlook is “remarkably similar” (2011, p. 21, fn. 30) to Dewey’s naturalism, noting too that it is unlikely that Anscombe ever read Dewey. Perhaps then it is not so surprising to find the 2012 Putnam drawn to Anscombe, if only in passing. In the rest of the paper, I make Anscombe’s philosophy of action central, specifically as a means of trying to establish in what respects only a Naïve Realism in perception can stem the sceptical worries that Putnam saw as originally flowing from the model theoretic arguments that his biv argument was supposed to stave off.

A materialist philosophy of action is Anscombian.^13^ I have so far suggested that a functionalist-geometric account of the semantics and use of ‘in’ is most at home with such a philosophy of action. Yet, as I insisted earlier, there is no reason to think that such an account (the functionalist-geometric position) should necessitate a particular philosophy of perception. We are now, I hope, in a position to see why it does. But to do so we need to treat carefully the *materialist* emphasis that the Anscombian philosopher of action recommends.

As the materialist rightly emphasises, the material with which we transact is typically intentionally-shaped—light switches, for instance, are designed to be switched on and off. This helps close the gap between action and the world. But in the case of perception, the gap is all too easy to re-insert, especially once an irrepressibly seductive conceptual picture of perception and our perceptual functioning remains in place. This picture is familiar. On this conception, conscious perceptual experience is primarily the upshot of the stimulation of our sensory surfaces by various worldly energetic inputs. Such inputs are informative of features and properties of the causally efficacious material world, aspects of which distinct features of conscious experience have evolved to represent. Space hardly appears on this conception at all. Or if it does, it only appears to the extent that the structure of a particular material landscape or environment structures the energetic input that the subject receives at her sensory peripheries. Emphasis only on material then, and on the structure of the material environment, may perhaps make it all too easy to omit or overlook explanatory consideration of the space *in which such material is*. This is why I suggest back-tracking to *Putnam’s Gesture*, just the kind of action beloved of the corporealist.

Now, naturally, larger action wholes like ‘going to the shop to buy butter’ also involve what I have called simple movement, such as my moving from the grocer’s homeward. I have also already noted that the concept of movement is part of the conceptual background that informs grasp of the human concept of ‘in’; it is characteristic of containers that they ‘exert’ locational control, be it strong or weak, on their containees. But since simple movement is sensitive to the structure of space, as we shall see, emphasis on the kind of movement that the corporealist emphasises—movement like *Putnam’s Gesture*—stands to uncover an important explanatory resource that might go overlooked if we focus only on the forms of human action or other forms of life that direct our attention only to material.

To bring out the explanatory significance of the structure of space, return to Ford’s reflection on the contours of bodily action quoted earlier. The body is said to take the shape, liquid-like, of whatever it acts upon but nothing is said of movement in space that we may be apt to describe as empty (say on a windless day). Ford does, however, note an important and, for my purposes, explanatorily relevant *disanalogy* between the movement of liquid poured from a height say, and the intentional movement of a body:

The most important disanalogy between the movement of the water and the movement of one’s hand is that, between the lip of the pitcher and its contact with an object, the water falls in the same way no matter what it falls upon. By contrast, the movements one makes in reaching out for an object differ according to the grasp that one must ultimately take of it.

That is so. Still, there is a commonality too. In neither case, in moving through an otherwise empty region, is the water nor the hand obstructed; there is no resistant material present. Yet that is not all. Part of the reason why the water falls the same way “no matter what it falls upon” is because the space it moves through is homogeneous in a sense I now detail. But the same is true, in this limited respect, of our *own movement* through space even if the particular movements we make in moving through space vary. I explain this thought by way of some historical (elementary) philosophy of physics.^14^

In 1876, in the newly established philosophical periodical *Mind*, Hermann von Helmholz argued that solid things are only freely mobile in spaces of constant curvature. By emphasising the possibility of free movement, he hoped to ‘prove’ that space is Euclidean, a proof which we now know to be invalid—the space in our vicinity is non-Eucildean.^15^ Still, we can dissociate the insight behind his production of the proof, from the proof itself.

**Fig. 3:**
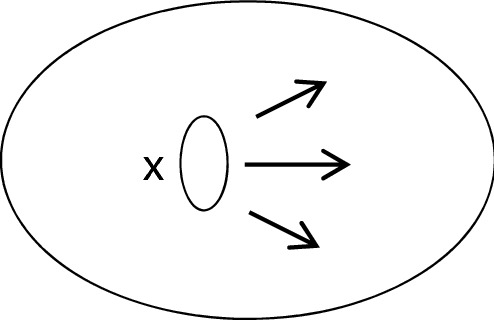
Adapted from Nerlich (1994, p. 83)

Take a space, such as the egg-shaped surface below, and a patch of paper, *x*, on that surface. If the patch were to move across the entire surface it would have to change shape; it would have to wrinkle to fit the tapered ends, or tear in cleaving to the widest part. Accordingly, to the extent that it would have to change shape in order to move throughout the space, we might say that it cannot move ‘freely’ through the space (Fig. 3).

Now, Helmholtz argued that ‘free’ motion, so understood, is a transcendental condition on the very intelligibility of the axioms of geometry. Helmholtz’s discredited proof notwithstanding, such axioms are intelligible to us. But this is not all. As Graham Nerlich emphasizes (1994, p. 83), “our *experience* of how things move in space suggests they are very freely mobile”, including our bodies.^16^ Nerlich’s thought here is that our bodies don’t have to change shape to move through spaces of different curvature so as to ‘fit’ into those spaces—not noticeably at least. We don’t experience the ‘rheumatism’ he imagines when he observes:

Since we ourselves are reasonably elastic we could move about in a space of variable curvature, but only by means of distorting our body shapes into non-Euclidean forms. We would have to push to get our bodies into these regions, for only forces will distort our shapes. If the curvature were slight, the rheumatism might be might be bearable and easy (p. 39).

Yet that this is so can obscure a simple—and if I am on the right track—explanatorily relevant fact. The spaces that we move through are homogenous in their curvature. But this in turn explains why we might be theoretically apt to overlook the sensitivity of movement to the structure of space, where this applies to not only the simple movement of our bodies, but to hoeing, stuffing, pouring, and the whole continual unfurling of dynamic human reality at large. For now, I focus only on the significance of the sensitivity of simple movement to the structure of space.

As Nerlich points out, space explains *non*-*causally*. For instance, when I move through space,

I don’t pressure the space and it doesn’t pressure me. I can’t push, pull or twist it; nor can it do that to me (ibid., p. 40).

That is to say, it is not in virtue of anything that space *does*, in an efficient sense, that explains why I can move through it this way rather than that. Rather, what explains the possibility of my moving through space, quite apart from the absence of resistant material located there, is the structure or shape of space itself, much as the shape of the egg-shaped surface explains why the patch can’t move freely around it. Space itself exhibits locational control. And, for Nerlich, since such explanation appeals to the topological and geometric properties of space, space explains *geometrically*, not causally.

Now, if this is right—without needing to commit to the fine-grained metaphysics that might be supposed to make any such claims true—and if free movement is explanatorily relevant to a grasp of what ‘in’ expresses in this way, we can, I think, finally, if still somewhat dimly, begin to see why focus on the locative rather than casually-mediated reference to brains and vats may, in the end, prove challenging for friends of envattedness. The way in which space thus explains is not causal, at least in an efficient sense. Yet the most that the seductive picture of perception can countenance from a theoretical perspective is to appeal to the structure of *input* at the subject’s periphery. But accordingly, a gap can be re-inserted between such input and the nature of the space she is in. And when the periphery of the subject is little more than a brain in a vat, the world outside that periphery can become as unrecognisible as you like.^17^

## Re-arrangement

The foregoing appeal to the structure of space—and geometric explanation—is I hope enough to scatter some seeds of doubt concerning the real possibility of what the biv scenario imagines. Yet it might well be objected that an analogue of locational control *can* be introduced in the vat. Why not allow that some computational constraint could mirror the constraint that the geometry of physical space places on our movement? Such a constraint would likewise be non-causal and structural—and it would not count as an “input”. All the biv advocate needs to do is to embrace the required semantics and allow non-causal influences to play semantic roles for certain terms.^18^ I have two responses to this suggestion; the first more or less concessionary, the second less so.

On the first, in the biv scenario, unlike our own, we should never have any access to the *grounds* of what non-causally but structurally influences our movement, and thereby our thought and talk. That is the cost of the concessionary move. The deeper point concerns the very *intelligibility* of the biv conceit.

In the passage quoted from MGF Martin earlier, he observes how the “the rearrangement of objects” in a space can reveal what was once obscured, say the face of a friend, hidden from view by tulips on the kitchen table. The rearrangement of objects in a space experienced does not involve an alteration in that space.

The argument I am sketching suggests that while space cannot be rearranged, its structure is relevant to an explanation of what grounds or makes possible any kind of rearrangement *in it*, assuming here that rearrangement involves movement in space. But, as such, the structure of space is, one might think, relevant too to an explanation of the possibility of the intelligibility of the concept of *re*-*placement*; of one thing taking *the place of* some other. Yet crudely speaking, this is what the biv scenario asks us to envisage, at least once experience is reified as some thing or event, the causes of which might be supplanted for one the other. For much as furniture can be rearranged in a room, it might be assumed that the philosopher can rearrange the universe such that things can seem to me now to be a certain way (the window beside my desk reveals an Autumn day), even where the world in which I am *really* now stranded is radically different (the acorns* and their oaks*). But if this is so, space having the structure it does, where things can be moved through it and re-placed, might be looked to as partly explanatorily of the very intelligibility of the biv fiction itself. It has a natural history. We are used to rearranging furniture, replacing things according to our purposes. Why not, in imagination, the causes of our experience and indeed our experiences themselves, like for like?

Granted a whole lot more work is needed to follow through on this speculation. For now, I think I can deliver simply on the question as to why any of this should bear on which philosophy of perception we should prefer.

Space cannot ‘get into experience’ on any account of perceptual experience that inserts the kind of gap that a materialist philosophy of action closes. I have shown that the shape of space is explanatorily relevant to an account of conditions on the grasp and intelligible use of ‘in’ but this feature of space—its structure—is not a causally efficacious property. Further, geometric explanation—the way space explains—applies to things *in* space, including agents stuffing suitcases. There is no path that connects an agent to the space that she is in. There is hence no path along which energetic inputs can travel before being intercepted by some sensory surface. Since Naïve Realism does not insert a gap, there is no requirement for space to ‘get into’ experience. Experience *involves* space and its material. And likely, though I have not defended this thought here, we get to think of things as being ‘in’ space in much the way that we come to treat of tulips in vases, though only the latter can be rearranged.

## An Objection

This appeal to space so as to do anti-sceptical work is not altogether new. It echoes an observation that Adrian Moore makes in an important paper. While Moore insists that “not much scepticism is required to think that the topology of space-time is left undetermined by what I *know* (Moore 1996, p. 222). where here he means, presumably, the global topology of space-time, he also writes that the *very possibility of scepticism*, the fact that I *can* conceive of full range of worlds compatible with my experience:

…. is not just due to the fact that the biography of my brain is part of the actual world, nor yet to the fact that it is part of that world and of others. It is due to the fact that I have thoughts that are suitably linked, through the range of capacities and dispositions that I have in virtue of possessing a normal body, *to different parts of space*-*time.* This cuts deeper than the fact that I have thoughts that are suitably sensitive to what trees are like. *If my thinking lacked this connection with space*-*time, then it would be out of touch with its own most fundamental nature* (my emphasis, p. 228)

As I likewise have urged, just as there is no gap between the apples and fields with which we transact, there is none between us the spaces we are in and move through; our connection with space-time is as intimate as could be. It might be objected, however, that I have everywhere appealed to movement in space where in fact the biv conceit demands only *experience* of movement in space. There need be no space, nor indeed any movement going on.

For instance, in the passage which introduces Putnam’s volitionism which I requoted above. It might be thought that what is needed to secure the appeal to movement is only seeings and feelings *as of* a hand being raised say. But while we might be tempted to allow that we cannot cleave apart the contours of bodily movement from the *objects* acted on, this thought assumes that we *can* skim off seeings and feelings from the things and events that they are putatively of or represent (and that can thereafter be coordinated by the murkier vat machinations). This, I suggest, is where the details of Putnam’s parenthetical appeal to Anscombe become potent, even if, as I show, aspects of his exegesis themselves need replacing.

Anscombe notes that sensations of movement are *non*-*seperable*. As I will cast things, this is a feature of their *form.* She explains the notion of non-separability with an example:

When I say: “the sensation (e.g. of giving a reflex kick) is not separable” I mean that the internal description of the ‘sensation’ – the description of the sensation-content – is the very same as the description of the fact known; when that is so, I should deny that we can speak of observing that fact by means of the alleged sensation (my emphasis, 1981, p. 72)

here she means that the internal description of the sensation gives a description of what one non-observationally knows—that one’s foot has moved say. One does not, that is, *infer* that one’s foot has moved on the basis of, or by means of, the sensation of giving a reflex kick, for this would be to suppose that the sensation can be *separately* described. What does this mean?

When considering an expression of the form “sensation of *X*”—say “the feeling of a hand being raised”—Anscombe says that we need to ask whether “of *X*” is a description of the sensation content or whether the sensation has some *other* content and *X* is what produces or goes along with the content (ibid.). She offers an example. Take the sensation of “going down in a lift”. “Going down in a lift” is an *external* description of the sensation but the experience can be characterized *internally* too—as “a sudden lightness”, “one’s stomach lurching upwards”.

Now, where such experiences can be separately described, this would seem to allow for a characterization of the experience as one whereby the external event in terms of which the experience is externally separately described is *productive of* the internal sensation *with which* the external event *goes along*. But consider now the feeling of raising one’s arm. This feeling is *not* separately describable. It is not conceived as produced by an *external* event—a hand-raising—*with which* an internally describable sensation ‘goes along’. Hand-raising is not ‘external’ to one, volitionism notwithstanding. We are in space.

A further point. Anscombe recognizes purely intentional uses of sensation verbs as well as material uses, as she calls them. A purely intentional use is one where a sensation verb like ‘see’ or ‘hear’ is *intended to be used* to characterize only how things may seem to one or strike one on some occasion. She lists a number of cases.

1. “I hear a ringing in my ears”.
2. “When you screw up your eyes looking at a light, you see rays shooting out from it”.
3. “Do you know how a taste can sometimes be quite indeterminate until you know what you are eating?” (1981, p. 12).

Yet while she recognizes uses such as (1)–(3), the *primary intended use* of verbs of sensation is, she says, *material*. The descriptions that are the direct object of the sensation verb are typically primarily intended as descriptions of things in the world that would be available for anyone to see (say) as such.

Now, viewed in an Anscombian light, the biv advocate might be said to go awry on two, related counts. Though sensations of movement are non-separately describable, we may, on occasion, undergo experiences that are like sensations of movement though in the absence of our having moved—say when a train on an adjacent platform pulls away. In many such cases, we may be apt to describe the sensation felt comparatively, as *like* the feeling of movement. Such a characterisation is intentional—it characterises how things strike one, how it feels to one. But this does not mean that there is a feeling of moving that is, after all, seperably describable. It remains the case that the descriptions that one comparatively deploys are those the form of which is non-seperable.

Now, *prima facie*, the BIV advocate might seem to overlook this fact. Rather, given the relative familiarity of movement-like experiences in the absence of movement, he might think not only that experiences of movement are fundamentally internal but that they are thereby separably describable; fundamentally internal since it might seem that *they* are produced by external events *with which they go along*; and separably describable since it seems that they can be described independently of the events that happen when they occur—after all, different kinds of occurrences, it might be supposed, can bring them about. For instance, the stimulation of the muscle spindles of a stationary arm may bring about the feeling enjoyed when raising one’s arm.

Whatever feeling this is, however, we have no reason to call it experience of movement though it may feel akin to such experience. Call this alternative experience of movement*. And certainly if *experience* of movement is relevant to *our* grasp of the locative—something which I have left open—then the biv advocate should advance experience of movement* as a candidate alternative. But if so, we should, I think, hang a star too on ‘in’. But whether conceiving of oneself as in* a vat* is enough to bring the original conceit into view however, to make it conceptually available, I leave the reader to consider. As I hope is now clear, I am sceptical.

## Conclusion

In this paper, I have tried out a fairly bold tack. Turning away from causally mediated reference, I have sought to explore conditions on a grasp of the concept which the intelligible use of ‘in’ expresses, suggesting that focus on the locative does after all urge a move towards the natural or Naïve Realism embraced by the later Putnam.

The proposal I have developed appeals to the structure of space but also to our vital nature and to the material and intentional form of our lived environment; a world populated by purses, vases, jugs, rooms, apples, hoes and fields. These worlds are not at removes from each other. The difficulty is to bring them into explanatory synchrony, a difficulty I probably haven’t quite overcome. Still, I hope two lessons are plain.

I explained why the appeal to the structure of space should trouble friends of envattedness—space explains movement non-causally—but I also suggested that the materialist should still make room, as part of larger action-wholes, for movement through space of the sort that *Putnam’s Gesture* exemplifies. In particular, I introduced a name for a concept that we have an implicit grasp of but which, if I am right, may well evade the biv: *locational control*. The structure of *where* we find ourselves and other things can constrain and shape possibilities for action and movement, a form of explanation that applies as much to Moore’s breakfast bowl as to the deeper metaphysical recesses of the spaces it moves through. The anti-sceptical argument needs both movements.^19^

## Footnotes

1 For discussion of the self-refuting character of Putnam’s argument, see Button (2016).

2 To be clear, the BIV scenario we are considering is one in which every sentient creature has been eternally envatted.

3 Yet as Moore (1996) notes, while the BIV argument appears in the first few pages of *Reason, Truth and History*, it is only much later in the work that indeterminacy arguments (which flow from model-theoretic considerations) are introduced: “What the later indeterminacy arguments show is that reference is not determined by truth conditions. In other words, *given* the truth conditions of what I think, or what I say, there are endless different reference relations in terms of which they can be specified. What the premise of the vat argument is concerned with is what determines truth conditions in the first place. The sense in which I cannot have thoughts about trees without coming into suitable causal contact with trees is the sense in which my thoughts cannot have certain arboreal truth conditions unless my having those thoughts is sensitive to what trees are like” (1994, p. 223).

4 This paper was recently published in a festschrift for Ned Block - see Putnam (2019).

5 Linguists exploring locative expressions often emphasise relations between objects—after all, in using locative expressions, we are typically describing the relation of something to some other thing. As a counterpoint, most philosophers of location start from the assumption of substantivalism (e.g. Gilmore 2013). See also Parsons (2007).

6 For mention of the notion of ‘location control’ see Coventry and Garrod (2004, p. 13).

7 Thanks to a sympathetic reviewer for encouraging me to note this important point, though it is one I can’t explore in this paper.

8 Those interested in pursuing a brain-based account of our semantic competence with the locative ‘in’ might look to O’Keefe (2003). But there are difficulties with doing so, not least of all the fact that the activation of place-cells is anchored to an animal moving its environment, and secondly, the fact that the very configuration of grid cells is sensitive to environmental geometry. The relevance of movement will become plain as the paper progresses.

9 See especially Coventry and Garrod (2004), and also Coventry et al (1994). Compare Zwarts (2017), which also contains a recent review of the state of the art. For more on the distinction between ‘at’ and ‘in’, see Wesche (1985).

10 For formative discussion of ‘rich’ content, see Siegel (2010).

11 For discussion, see MacPherson (2011).

12 Note that ‘fill’ itself doesn’t specify a manner of motion.

13 For clear and lucid exposition of what this amounts to, see Wiseman (2016).

14 I present this thought in a highly intuitive and non-technical way. I’m grateful to an anonymous reviewer for helping me work out how to frame this appeal to the structure of space.

15 As Friedman (1997) points out, Helmholtz’s empiricism is misplaced in light of what relativity teaches—a change in the distribution of matter affects curvature. I leave this complication aside.

16 Intriguingly, for Helmholtz, our experience of bodily movement plays a special *epistemic* role. He observes: “as a small elastic flat disc, say of india-rubber, can only be fitted to a slightly curved spherical surface with relative contraction of its border and distension of its centre, so our bodies, developed in Euclid’s flat space, could not pass into curved space without undergoing similar distensions and contractions of their parts, their coherence being of course maintained only in as far as their elasticity permitted their bending without breaking (ibid. p. 15, para 318)” Adding optimistically: “we can *infer* from the known laws of our sensible perceptions the series of sensible impressions which a spherical or pseudospherical world would give us” (my emphasis, ibid.) Cf. the Ancombian position I sketch.

17 In particular, it can be radically spatially different to the world we take ourselves to be ‘in’. This is the assumption behind David Chalmers’ recent discussion of spatial functionalism. See Chalmers (2006, 2012 and 2019). See also Thompson (2010) and, for a critique, Epstein forthcoming. The whole current of the present paper runs counter to Chalmers’ proposal.

18 I am very grateful to a reviewer for raising this point and for offering this formulation of the objection.

19 I had a first attempt at setting out some of these ideas in Paris in 2015, at an NYU/IJN workshop on spatial functionalism. I also presented related material in Bergamo, Edinburgh and in Cambridge. I am grateful to everyone in attendance on those occasions but especially to David Chalmers, Brad Thompson, Coltilde Calabi and Alfredo Paternoster. I am very grateful to Tim Crane for funding to work on this and related material as part of his Templeton Cambridge New Directions in the Study of the Mind award. I plainly have a bit more work to do in this area! Thanks to Kim Davies for careful comments and general discussion of scepticism; to Tim Button for initial encouragement when I wasn’t really sure where I was going with this; to Craig French for his (as usual) brilliant comments on an earlier draft; and to Rachael Wiseman and Andy Hamilton for helping me to think myself out of the vat with help from Wittgenstein and Anscombe. Two illuminating reports for *Erkenntnis* led me to reshape this material in critical respects.
